# Genetic Identity, Diversity, and Population Structure of CIP's Sweetpotato (*I. batatas*) Germplasm Collection

**DOI:** 10.3389/fpls.2021.660012

**Published:** 2021-10-28

**Authors:** Noelle L. Anglin, Ronald Robles, Genoveva Rossel, Rocio Alagon, Ana Panta, Robert L. Jarret, Norma Manrique, David Ellis

**Affiliations:** ^1^International Potato Center (CIP), Lima, Peru; ^2^Plant Genetic Resources Conservation Unit, United States Department of Agriculture, Agricultural Research Service, Griffin, GA, United States

**Keywords:** sweetpotato, germplasm, genebank, population structure, genetic diversity, SSRs, genetic identity

## Abstract

The *in trust* sweetpotato collection housed by the International Center of Potato (CIP) is one of the largest assemblages of plant material representing the genetic resources of this important staple crop. The collection currently contains almost 6,000 accessions of *Ipomoea batatas* (cultivated sweetpotato) and over 1,000 accessions of sweetpotato crop wild relatives (CWRs). In this study, the entire cultivated collection (5,979 accessions) was genotyped with a panel of 20 simple sequence repeat (SSR) markers to assess genetic identity, diversity, and population structure. Genotyping and phenotyping of *in vitro* plantlets and mother plants were conducted simultaneously on 2,711 accessions (45% of the total collection) to identify and correct possible genetic identity errors which could have occurred at any time over the thirty plus years of maintenance in the *in vitro* collection. Within this group, 533 accessions (19.6%) had errors in identity. Field evaluations of morphological descriptors were carried out to confirm the marker data. A phylogenetic tree was constructed to reveal the intraspecific relationships in the population which uncovered high levels of redundancy in material from Peru and Latin America. These genotypic data were supported by morphological data. Population structure analysis demonstrated support for four ancestral populations with many of the accessions having lower levels of gene flow from the other populations. This was especially true of germplasm derived from Peru, Ecuador, and Africa. The set of 20 SSR markers was subsequently utilized to examine a subset of 189 accessions from the USDA sweetpotato germplasm collection and to identify and reconcile potential errors in the identification of clones shared between these collections. Marker analysis demonstrated that the USDA subset of material had 65 unique accessions that were not found in the larger CIP collection. As far as the authors are aware, this is the first report of genotyping an entire sweetpotato germplasm collection in its entirety.

## Introduction

Sweetpotato is an important crop globally as a versatile food source in that both the roots and the leaves can be consumed. The crop is also utilized in the manufacturing of industrial and food products such as starch, flour, noodles, colorants, candy, chips, and alcohol (Lebot, 2008), in addition to fodder and fish food. In 2018, over 91 million tons of sweetpotato were produced worldwide on over eight million hectares of land with China being the largest producer (FAO statistics, http://www.fao.org/faostat/en/#home). However, sweetpotato is also widely cultivated in many developing countries where the yields are well below the average, compared with developed countries. Sweetpotato is also an important source of income for women compared with men in developing countries, such as Nigeria, due to its low input requirements and short maturity time relative to other crops (David, 2015).

Sweetpotato also grows well on marginal soils with limited inputs and is tolerant to a wide range of climatic conditions making production improvements feasible (Lebot, 2008). Sweetpotato is a versatile food source that is nutritionally beneficial for human health and serves a medicinal role in many parts of the world. Leaves, stems, or raw roots are consumed for the treatment of type 2 diabetes in Ghana, inflammatory and oral infections in Brazil, anemia, and hypertension in Japan, and prostatis in Cameroon. Roots contain simple and complex carbohydrates, vitamins (A, B1, B5, B6, and C), minerals (iron, calcium, magnesium, manganese, and potassium), fiber, flavonoids, phenolics, and iron, while the leaves contain protein, fat, fiber, ash, carbohydrates, vitamins (A and C), minerals, and low levels of toxicants (phytic acid, cyanide, tannins, and oxalate) (Mohanraj and Sivasankar, 2014).

Vitamin A deficiency, a significant problem in many developing countries, contributes to 6% and 8% of deaths of children under 5 years of age in Africa and Southeast Asia, respectively, linked to inadequate intake of vitamin A or pro-vitamin A. This situation has persisted despite years of introducing supplements and fortification (Paul et al., 2017). Deficiencies in vitamin A also leads to significant problems in pregnant women in many countries throughout Africa and Asia (Rodriguez-Bonilla et al., 2014). Studies have shown that 3.5 ounces of sweetpotato root contains 100–1,600 μg of retinol activity (vitamin A), meeting 35–90% of vitamin A requirements (Mohanraj and Sivasankar, 2014). Thus, the high beta-carotene content in orange-fleshed sweetpotatoes is an affordable strategy to help combat this deficiency (Niringiye et al., 2014) in developing countries that are willing to adopt orange-fleshed sweetpotato into their diet.

Cultivated sweetpotatoes are classified as *Ipomoea batatas* in the family *Convolvulaceae*. The genus *Ipomoea* contains more than 600 species. Due to the large size of this genus, sweetpotato and its near relatives were grouped in Series Batatas which consists of 14 taxa. Cultivated sweetpotato is an allogamous species and is self-incompatible. Though, the majority of sweetpotatoes are propagated and disseminated as clones or *via* slips rather than from seed produced from deliberate crossing. The sweetpotato genome is characterized as a hexaploid (2n = 6x = 90). However, there are various reports of the existence of tetraploids (2n = 4x = 60) or feral *I. batatas* (Austin, 1988; Bohac et al., 1993). The existence of tetraploids is rare as most all cultivated sweetpotatoes are hexaploids. Because of the polyploidy nature of sweetpotatoes, Mendelian genetics and segregation ratios are quite complex (Lebot, 2008). The origin and domestication of the cultivated sweetpotato remain uncertain, although several hypotheses have been published providing support for various theories of its origin. Austin (1988) suggested that *Ipomoea trifida* and *Ipomoea triloba* were the prime candidates to be the closest relatives of cultivated sweetpotato and that *I. batatas* arose by unreduced gametes following hybridization of these wild species. Others have reported 2n pollen production of *I. trifida* and assessed the fertility of synthetic hexaploids leading to the hypothesis that sweetpotato may have arisen from hybridization and unreduced gametes between *I. trifida* and a tetraploid *I. batatas* (Orjeda et al., 1990; Freyre et al., 1991). Rouillier et al. (2013) suggested multiple origins evolving from two distinct autopolyploidization events from an ancestor cultivated sweetpotato shares with *I. trifida*. Recently, a report suggested that *I. trifida* was the sole progenitor of sweetpotato and that it had an autopolyploid origin (Munoz-Rodriguez et al., 2018). Other studies have proposed an allo-autohexaploid (2n = 6x = 90) origin with a *B*_1_*B*_1_*B*_2_*B*_2_*B*_2_*B*_2_ genome for sweetpotato which was produced by crossing a tetraploid and a diploid followed by whole genome duplication occurring ~341,000 years ago (Yang et al., 2017). Yang et al. (2017) also assumed the diploid progenitor of cultivated sweetpotato was *I. trifida* but reported the tetraploid progenitor was unknown.

The principal purpose of an *ex-situ* genebanks is to preserve and safeguard the genetic diversity of important crops and their wild relatives for use today and for future generations. Clonal genebanks, as opposed to seed genebanks, are unique in that accessions are genetically static with fixed combinations of alleles that are preserved over time vs. seed accessions which are mostly populations with alleles that are in flux. In either case, each accession in a genebanks potentially contains new alleles or a combination of alleles that confer specific traits and adaptive potential (McCouch et al., 2012). Access to this genetic diversity is a key component for breeders and related research activities to improve cultivated crops and ensure food security. Frequently, elite lines continue to be improved while the exotic lines remain untapped, uncharacterized, and underutilized (Lee, 1998). In general, genebanks strive to preserve as much of the genetic diversity as possible found in their mandated crops and associated wild relatives. Thus, collection sizes are often quite large, but only rarely are they thoroughly characterized for traits of interest. This limits the ability of the user to strategically select genotypes or phenotypes for their specific purpose. Consequently, only a relatively small proportion of holdings of the genebanks tend to be utilized in the short term, and most genebanks resources are utilized in maintaining accession viability and genetic integrity, and less so on understanding the specific genetic attributes and key traits of the accessions in the collections.

Categorizing *I. batatas* into varietal groups based on morphological traits has proven to be quite difficult because the variation is continuous (Roullier et al., 2011) and due to residual GxE affects. However, the decreasing cost of sequencing and genotyping services have made it possible to characterize a larger number of accessions or an entire genebanks collection(s) at the molecular level. For example, 784 landrace populations representing 799 accessions of maize from institutional collections were evaluated by simple-sequence repeat (SSR) markers to reconstruct patterns of diffusion (Mir et al., 2013). Inbred lines (1,191 accessions) from the INRA maize collection were analyzed with genotyping by sequencing (GBS) to evaluate genetic diversity and assign ancestral groups (Gouesnard et al., 2017). Over 2,000 accessions of apples from the UK National Fruit collection were evaluated with DArT markers to elucidate the diversity in the germplasm collection (Ordidge et al., 2018). The entire lettuce germplasm collection (2,323 accessions) at the Center for Genetic Resources (CGN) was characterized using three AFLP primer combinations (Van Hintum, 2003). More recently, a large collection of barley germplasm (21,406 accessions) from the Genebanks of Leibniz Institute of Plant Genetics and Research (IPK, Gatersleben, Germany) was genotyped by GBS to evaluate global population structure and domestication (Milner et al., 2019). It is easy to envision that in the future genebanks will become increasingly involved in managing and interpreting digital resources (whole-genome sequence data, high throughput marker data, image files, etc.) collected on entire collections and in which the user community will ultimately mine.

Various studies have been conducted to examine genetic diversity, to a limited extent, within the *I. batatas* gene pool. Rodriguez-Bonilla et al. (2014) utilized SSR markers to evaluate the genetic diversity of 137 Puerto Rico landraces. In that study, 255 alleles were identified at 21 loci with an observed heterozygosity of 0.71 across populations. Gwandu et al. (2012) examined diversity within 57 Tanzanian elite sweetpotato genotypes using four SSR markers. The results showed no specific clustering based on disease, dry matter content, or geographic location. The data was highly variable with 395 alleles produced. Wadl et al. (2018) examined a portion of the USDA sweetpotato germplasm collection (417 accessions) *via* GBS. The data analysis revealed four subpopulations within the collection, with high levels of mixed ancestry. Phylogenetic and principal components analysis (PCA) supported the population structure analysis.

The genebanks at the International Potato Center (CIP) in Lima, Peru holds one of the largest *ex situ* collections of sweetpotato germplasm containing almost 6,000 accessions of cultivated *I. batatas* held *in trust* under the International Treaty for Plant Genetic Resources for Food and Agriculture (ITPGRFA). This collection includes material obtained from 63 countries and consists of landraces, breeding lines, and improved varieties. A large portion of the material in this collection originated or was developed in Peru. These clonally propagated materials are distributed to requestors worldwide as phytosanitary cleaned *in vitro* cultures for research, breeding, and education. Originally, plant materials were maintained in the field. However, this resulted in multiple problems including exposure of accessions to pests and disease, variable environmental conditions, loss of accessions due to attrition, and mixing of accessions due to mislabeling, mishandling, or contaminating volunteer plants. The procedures used for maintenance were complex and expensive. These, and other factors, led to concerns regarding the identification and adoption of appropriate maintenance techniques (Huaman, 1999; Jarret et al., 2019). Therefore, clonally propagated materials have been maintained since the 1980s as *in vitro* cultures. This strategy provides long-term security, easier routine maintenance, and conservation in disease and pathogen-free state that facilitates distribution and use.

The objectives of this project were to (i) evaluate the genetic identity and diversity of all accessions within the CIP sweetpotato collection using a panel of 20 SSR markers, (ii) evaluate phylogenetic relationships and population structure of the *I. batatas* collection, (iii) identify potential duplicates in this collection, and (iv) compare the CIP collection with a subset of sweetpotato accessions from the USDA genebanks.

## Materials and Methods

### Plant Material and DNA Extractions

Young leaves from either greenhouse-maintained plants or *in vitro* cultures were collected from 5,979 accessions from the CIP genebanks (Supplementary Table 1). DNA was extracted using the CTAB method according to Doyle and Doyle (1990) with modifications. Approximately, 100 mg of leaf tissue was collected in an Eppendorf microcentrifuge tube containing 700 μl of CTAB extraction buffer (2% CTAB, 20 mM EDTA pH 8, 1.4 M NaCl, 10 mM Tris-HCl pH 8, 1% PVP, and Milli-Q® water) along with 2 μl of β158 mercaptoethanol. Ceramic beads were placed in the microcentrifuge tubes and the leaves were ground using a FastPrep®-24 (MP Biomedicals, SA, CA, USA) for 1 min. The resulting homogenate mixture was incubated for 45 min at 65°C, and then 750 μl chloroform: isoamyl alcohol (24:1) was added. The tubes were then gently inverted for 5 min and centrifuged at 14,000 rpm for 5 min at room temperature. The aqueous phase was transferred to a new tube containing 70 μl of 10% CTAB buffer (10% CTAB, 20 mM 163 EDTA pH 8, 0.7 M NaCl, 10 mM Tris-HCL ph8, and Mili Q water). Chloroform: isoamyl alcohol [24:1] (750 μl) was added to this mixture and centrifugation was repeated to separate the layers. The upper phase was transferred to a new tube to which an equal volume of cold isopropanol was added and incubated at −20°C for 15 min. The tube was centrifuged at 14,000 rpm for 5 min and the pellet was washed with 500 μl of 70% ethanol. The pellet was air-dried and resuspended in 100 μl of TE buffer pH 8 into which 1 μl RNAse (10 mg/ml) was added prior to incubation at 37°C for 2 h. The quality and quantity of the DNA were determined by running 1 μl of the resuspended DNA solution on a 1% agarose gel with lambda DNA as a standard and also by measuring the DNA concentration on a NanoDrop 2000 Spectrophotometer Thermo Scientific (Nanodrop Products, DE, USA). The DNA was diluted to 20 ng/μl for Polymerase Chain Reaction (PCR) amplification.

Leaf material harvested from *in vitro* cultures of accessions in the USDA sweetpotato germplasm collection (Supplementary Table 2) were collected and DNA was extracted using the Qiagen (Hilden, Germany) DNeasy Plant Mini 175 Kit following guidelines of the manufacturer. The DNA extracts were subsequently shipped to Lima, Peru for genotyping and analysis.

### PCR Amplification and Allele Separation

A panel of 20 SSR markers was selected for this project (Table 1). All forward primers were tailed with M13 sequence for universal fluorescent dye labeling for detection on a polyacrylamide gel attached to an LI-COR IR2 4300 Global DNA analyzer (LI-COR, Lincoln, NE). Primers were multiplexed when possible but, in some cases, it was necessary to amplify primers individually (Table 1). The PCR reaction consisted of nuclease-free water (Sigma-Aldrich, St. Louis, MO, USA), PCR buffer which included the MgCl_2_ (Promega, Madison, WI USA), unlabeled M13 tailed forward and reverse primers (Integrated DNA Technologies, Coralville, Iowa), Taq polymerase (Promega, Madison, WI USA), dNTPs (Invitrogen Carlsbad, CA), universal M13 IRD 700/800 fluorophore dye label primer (Integrated DNA Technologies), and 10 ng/μl final concentration of template DNA. The total reaction volume per individual PCR reaction was 10 μl. Specific details of the final concentration of each component and primer combinations for multiplexing are listed in Table 1. Thermocycling conditions consisted of one cycle at 94°C for 4 min, followed by 31 cycles of 94°C for 1 min, 60–62°C for 1 min (depending on the primers utilized), 72°C for 1 min, followed by 1 cycle of 72°C annealing step for 10 min, and then held at 4°C. All PCR reactions were performed in a Veriti® 96-Well Thermal Cycler from Applied Biosystems (Waltham, Massachusetts).

**Table 1:** List of sequences for the 20 Simple Sequence Repeat (SSR) markers employed in this study.

**Marker Name**	**Forward Sequence**	**Reverse Sequence**	**Multiplexing**	**Annealing Temp (C)**	**F** **(μM)**	**R** **(μM)**	**PCR 10X Buffer**	**dNTPs (mM)**	**M13 tail IRD700 (μM)**	**M13 tail IRD800 (μM)**
IBS11	CACGACGTTGTAAAACGAC CCCTGCGAAATCGAAATCT	GGACTTCCTCTGCC TTGTTG	A	62	0.016	0.024	0.93X	0.165	0.023	–
IBS141	CACGACGTTGTAAAACGAC GAAGCAGTAGTTGTGTTGC TTT	CTCTATCTTTATCTC TTCCGGC	A	62	0.015	0.0225				
IBS199	CACGACGTTGTAAAACGAC TAACTAGGTTGCAGTGGTTT GT	ATAGGTCCATATAC AATGCCAG	A	62	0.016	0.024				
IbJ116A	CACGACGTTGTAAAACGAC TCTTTTGCATCAAAGAAATC CA	CCTCAGCTTCTGGG AAACAG	B	61	0.02	0.03	0.93X	0.2	–	0.038
IBE2	CACGACGTTGTAAAACGAC CAGCCGCCAAGTTTTCTACA	AGGCGGAGGCTGATAATGA	B	61	0.017	0.0255				
IBS28	CACGACGTTGTAAAACGAC ATATCTTCCAACAGTCTGCC TT	GCTTTCTGCTCTTC TTTCACTT	C	61	0.029	0.0435	0.93X	0.165	–	0.045
IBC12	CACGACGTTGTAAAACGAC TCTGAGCTTCTCAAACATGA AA	TGAGAATTCCTGG CAACCAT	C	61	0.017	0.0255				
IbJ522a	CACGACGTTGTAAAACGAC ACCCGCATAGACACTCACCT	TGACCGAAGTGTA TCTAGTGG	D	60	0.037	0.0555	0.93X	0.165	–	0.04
IBS14	CACGACGTTGTAAAACGAC ATCAGACATGCTTTTGTGAG AC	AGGGACTCACTTTC ACTGCTAT	D	60	0.01	0.015				
IbJ544b	CACGACGTTGTAAAACGAC AGCAGTTGAGGAAAGCAAG G	CAGGATTTACAGC CCCAGAA	E	60	0.027	0.0405	0.93X	0.165	n/a	0.037
IBS24	CACGACGTTGTAAAACGAC AGTGCAACCATTGTAATAGC AG	TCCTTTCTTCATCAT GCACTAC	E	60	0.02	0.03				
IBS139	CACGACGTTGTAAAACGAC CTATGACACTTCTGAGAGGC AA	AGCCTTCTTGTTAG TTTCAAGC	F	61	0.012	0.018	0.93X	0.165	0.033	–
lbY44	CACGACGTTGTAAAACGAC CAAGAAGAGCATAAGCGTG AGAT	GCGATCTGAGAAG GTGATAATTG	F	61	0.01	0.015				
1B544	CACGACGTTGTAAAACGAC CAAGAAGAGCATAAGCGTG AGAT	TTCATTGTGACTGT GAGGAAG	F	61	0.016	0.024				
lbJ1809	CACGACGTTGTAAAACGAC TTAATACACATGCCTCTCCA TC	GATAGTCGGAGGC ATCTCCA	G	60	0.023	0.0345	0.93X	0.165	0.037	–
1BY60	CACGACGTTGTAAAACGAC CTTCTCTTGCTCGCCTGTTC	GCGTTTTACAAGAT TCAGAAACCAC	G	60	0.015	0.0225				
lbJlOa	CACGACGTTGTAAAACGAC TCTCTCTGTTATGTTATGGT GATGG	GTAATTCCACCTTG CGAAGC	H	60	0.02	0.03	IX	0.165	0.03	–
1B530	CACGACGTTGTAAAACGAC TCAACCACTTTCATTCACTCC	CCTGTATTTCCACA ACCTACAA	I	62	0.022	0.033	0.93X	0.1665	0.033	–
1B582	CACGACGTTGTAAAACGAC GGTCCTGTTAAAACAGCTCC TA	GAAATGGCAGAAT GAGTAAGG	J	60	0.018	0.027	0.93X	0.1665	0.03	–
1B286	CACGACGTTGTAAAACGAC GACATAATTTGTGGGTTTAG GG	GGTTTCCCAATCAGCAATTC	K	60	0.02	0.03	0.93X	0.1665	0.03	–

*Universal M13 tails were added to the forward sequence to reduce the cost of individually labeling each primer set with a fluorescent dye. The PCR annealing temperatures and final concentrations of each component in the reaction are listed above. Markers with the same letter designation were multiplexed together in a single PCR reaction. When possible, markers amplified individually were pooled together prior to loading on the polyacrylamide gel*.

The PCR products were separated using a 5.5% polyacrylamide gel (KBPlus Gel Matrix, LI-COR) attached to an LI-COR IR2 4300 Global DNA Analyzer dual dye system (LI-COR, Lincoln, NE). The PCR products were mixed with 5 μl of Blue Stop Solution (LI-COR, Inc. Lincoln, NE) and then denatured (95°C) for 3 min and immediately chilled on ice prior to gel loading. Approximately, 0.2 μl from each PCR product was loaded into each gel lane. A 50–350 bp size standard (LI-COR, Lincoln, NE) was loaded into the gel at lane positions 1, 10, 19, 28, 37, 46, and 55 of each gel. Gel images were visualized with the SAGA^GT^ software version 3.2 (LI-COR, Lincoln, NE). Alleles were scored using a binary code (0/1) for the presence or absence of fragments. Missing data were coded as “9” (All data produced from the SSR markers can be viewed in Supplementary Table 3). Markers were scored in this fashion because sweetpotato is a hexaploid and allele dosage cannot always be determined (i.e.,—ABBBBB, AABBBB, AAABBB, and AAAABB appear the same).

### Data Analysis

Most all programs created for data analysis and genetic population studies have revolved around haploids and diploids, making polyploids a challenge to analyze (Dufresne et al., 2013; Meirmans and Liu, 2018). Inheritance patterns can be variable within individual genomes leading to a disomic and polysomic inheritance which greatly complicates genetic analysis because they assume a specific mode of inheritance and in polyploids mixtures of inheritance occur (Dufresne et al., 2013). Sweetpotato is known to have a mixture of inheritance patterns and it is not clear if it is a pure autohexpaloid, allohexaploid, or an allo-autohexaploid, which further complicates the analysis. Scientists, therefore, have frequently “diploidized” the molecular data because it was not possible to determine allele dosage, model the complex inheritance patterns that vary across the genome, and the programs available for analysis were created for diploid species.

Presence-absence data was utilized to construct phylogenetic trees. A genetic dissimilarity matrix among accessions was calculated using the Jaccard coefficient in Dissimilarity Analysis and Representation for Windows (DARwin) v6.0.14 (Perrier and Jacquemoud-Collet, 2006). Trees were constructed using the unweighted Neighbor-Joining method in the DARwin package.

Phylogenetic trees were annotated using Interactive Tree of Life (iTOL) (Letunic and Bork, 2016) to color code branches by country of origin. Principal Coordinate Analysis (PCoA) was carried out by using DARwin (version 6.0.14) software based on Jaccard's distance.

The PIC values were calculated using the R package polysat (Clark and Jasieniuk, 2011) utilizing the following formula developed by Botstein et al. (1980):


$$\begin{array}{l} 1-\left(\underset{i=1}{\sum }p^{2}i\right)-\overset{n-1}{\underset{i=1}{\sum }}\underset{j=i+1}{\sum }2pi^{2}pj^{2} \end{array}$$

Population structure for the dataset was estimated using the program STRUCTURE v2.3.4 (Pritchard et al., 2000). This program infers population structure and assigns individuals to populations based on their genotypes. STRUCTURE uses model-based clustering in which a Bayesian approach is used to identify clusters based on a fit to a Hardy-Weinberg equilibrium and linkage equilibrium. Multiple runs of STRUCTURE were performed by setting K (the number of populations) from 1 to 10 and producing 10 replications for each K. The burn-in time was set to 10,000 and MCMC replications to 100,000. The ancestry model was set to admixture and allele frequencies were correlated. Because finding an optimal K value for data sets can be a challenge. The Evanno method (Evanno et al., 2005) was employed in Structure Harvester (http://taylor0.biology.ucla.edu/structureHarvester/) to choose the optimal K value (Earl and vonHoldt, 2012). In addition, statistical analyses generated from STRUCTURE runs including consistency in alpha during the run, a proportion of individuals not being symmetric (~1/K), and a low Pr(X/K) value was also employed to determine an appropriate K value.

## Results and Discussion

### Diversity and Population Structure of the Sweetpotato Germplasm Collection

The SSR genotyping was carried out on a total of 5,979 accessions from the CIP sweetpotato germplasm collection which consisted of 4,255 landraces, 1,470 breeding lines, 236 improved varieties, and 18 non-classified accessions collected or donated from 63 countries worldwide (Supplementary Table 1). The country with the highest proportion of accessions was Peru (27.3%), followed by Nigeria (14.60%), Papua New Guinea (6.5%), Taiwan (5.10%), and Indonesia (5%). Accessions native to Latin America were largely collected through numerous collection expeditions starting in 1985 and through donations from national collections (Valle Grande Institute Ica, Peru; Lambayeque University Ayacucho, Peru). Accessions from Africa, Asia, and the Pacific were mostly transferred *via* a donation from the following collections: the International Institute of Tropical Agriculture (IITA) in Nigeria, the Asian Vegetable Research Center (AVRDC) in Taiwan (now The World Vegetable Center), and the CIP regional office in Kenya.

**Figure 1:**
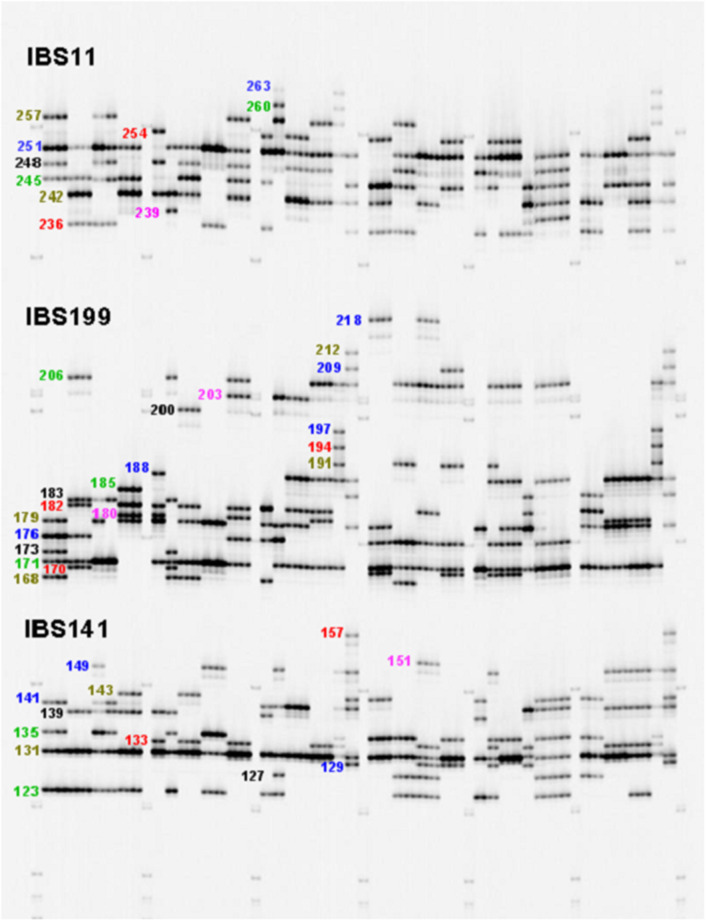
LI-COR electrophoresis gel image for three SSR loci (IBS11, IBS199, and IBS141) that were multiplexed in a single reaction, with approximate allele sizes.

**Figure 2:**
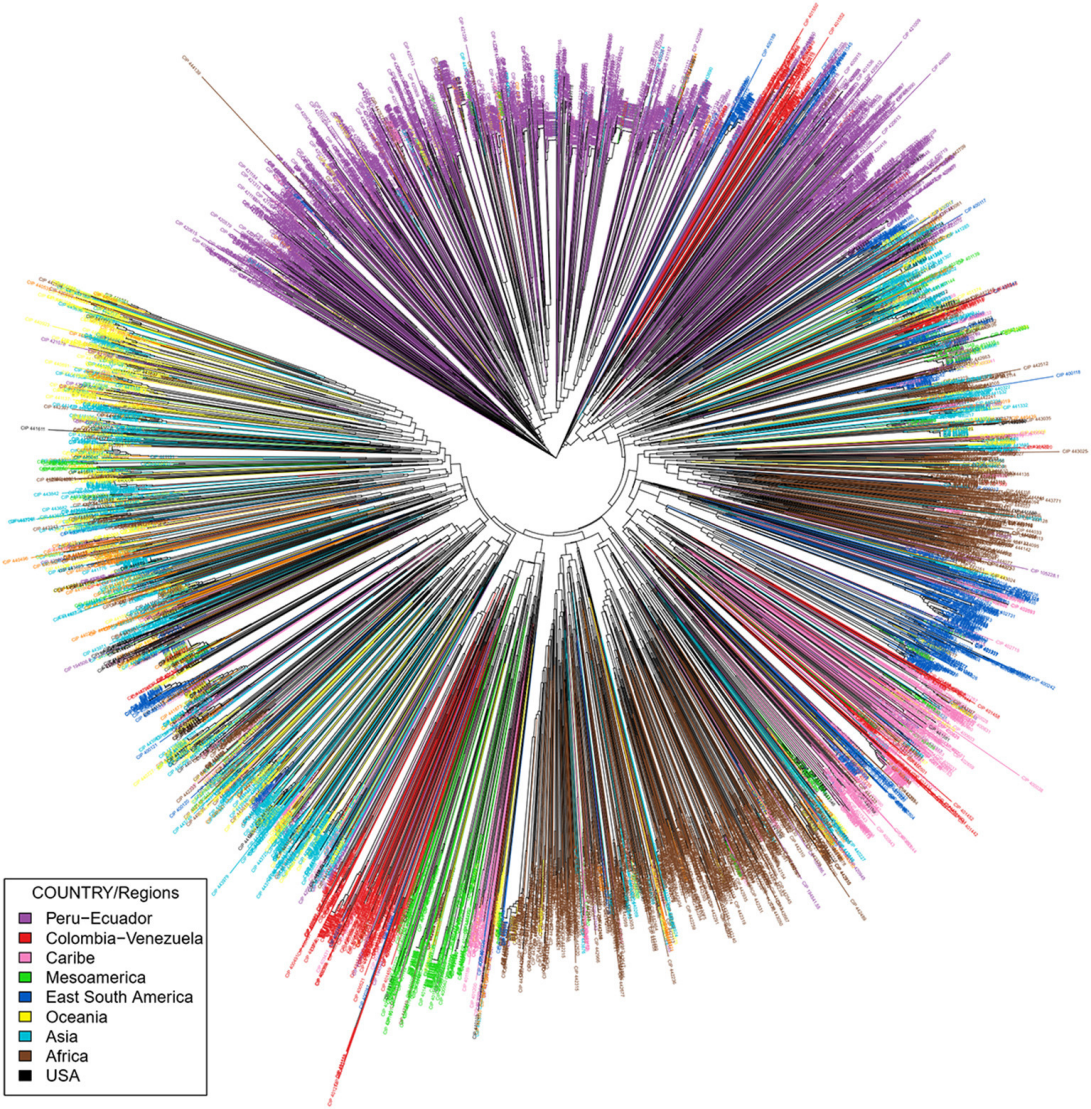
Phylogenetic tree constructed with Neighbor Joining (NJ) of 5,979 sweetpotato accessions from the CIP germplasm collection. iTOL was employed to color code branches by country of origin or donation country. Peru = purple. Red = Venezuela and Colombia. Green = Mexico and Central America. Pink = Caribbean. Africa = brown. USA = black. Blue = Argentina, Paraguay, and Brazil. Turquoise = Asia. Yellow = Oceania.

Details of the 20 SSR markers chosen to assess genetic diversity and population structure are presented in Table 1. Multiplexing was employed for several of the SSR markers for cost savings and efficiency. Two dinucleotides, four tetranucleotides, and 14 trinucleotide markers were utilized (Table 2) in this study. These markers were chosen based on polymorphisms and the production of clear scorable, reproducible alleles in sweetpotato avoiding markers with stuttering (Alagon, unpublished data). The number of alleles per marker ranged from 5 to 27 with an average of 14.85 alleles/locus. An example of the allelic diversity observed is shown in Figure 1. The polymorphism information content (PIC) which is a measure of informativeness of a given marker ranged from 0.5180 to 0.883 with an average of 0.7816 per marker for the sweetpotato germplasm at CIP (Table 2). Marker data was used to create a phylogeny to examine intraspecific relationships among all *I. batatas* genotypes. Phylogeographic signals were apparent in this data set in which many accessions clustered according to geographic location (Figure 2). Principal Coordinate Analysis (PCoA) was also performed to assess the variation in the entire collection (Supplementary Figure 1) which effectively reduces the dimensions of the dataset and displays the range of variation. Peru-Ecuador (purple) and Colombia-Venezuela (red) can be distinguished more clearly than the others which were also observed in the phylogeny. The percentage of variation in the first component was 6.7%, and 2.44% in the second component suggesting low genetic variance in this material. This low range of genetic variance could be due to redundant germplasm as can be seen by a plot of all the pairwise genetic distances which demonstrates the genetic redundancy (Supplementary Figure 2). Overall, branch lengths between major groupings in the phylogeny were short suggesting minor genetic variation among clades and/or an extensive amount of hybrid accessions within the collection. Low genetic variation was also observed in the PCoA analysis with little variation explaining the first and second components of this data set. This low genetic divergence in the accessions may be due to the number of markers used in this study or because allele dosage which could not be inferred in this study with any great confidence due to the ploidy of the samples, potentially producing less genetic variability in the dataset. Due to this concern, all accessions were morphologically characterized for 30 descriptors, had their passport data compared with accessions with similar fingerprint patters, and all of these data were combined together and compared leading to a more robust analysis.

**Table 2:** Summary of SSR markers utilized in the current study including the total number of alleles, polymorphism information content (PIC) value, motif type, and allele sizes as detected in sweetpotato.

**Primer**	**Allele sizes** **(Base Pairs bp)**	**Total number of alleles**	**Mean # of alleles per individual**	**PIC** **(calculated with 5979 CIP accessions)**	**PIC** **(USDA material)**	**Motif**
IBS11	224–278	15	3.42	0.8113	0.8155505	(TTC)10
IBS141	123–157	17	4.06	0.8688	0.8375046	(CTTT)6
IBS199	168–218	26	4.00	0.8616	0.8530219	(ACA)7
IbJ116A	202–265	19	3.42	0.7933	0.7983935	(CCT)7
IBE2	106–166	20	2.98	0.8695	0.8453308	(TCT)13
IBS30	189–250	20	3.50	0.8114	0.7948903	(ATT)9
IBS82	138–163	15	3.50	0.8172	0.8128695	(TCA)7
IB286	105–134	10	3.2	0.8408	0.8139889	(CT)12
IBS28	180–246	24	3.6	0.8657	0.8278914	(TTC)8
IBC12	111–147	11	3.4	0.8028	0.8187527	(TTC)6
IBS139	301–354	27	2.89	0.8446	0.85462	(GA)7
IbY44	183–219	13	3.62	0.8023	0.8079592	(AGA)6
IBS44	127–130	12	3.69	0.8159	0.8068511	(ATC)8
IbJ10a	177–224	12	2.82	0.8123	0.8295309	(AAG)6
IbJ522a	223–268	9	2.59	0.6500	0.602318	(CAC)7
IBS14	188–212	5	2.64	0.5180	0.5073643	(TATG)7
IbJ1809	137–158	8	2.87	0.7264	0.734734	(CCT)6
IBY60	188–225	14	3.14	0.7782	0.7717795	(TAT)5
IBS24	145–181	12	2.89	0.7320	0.7230113	(CTGC)7
IbJ544b	191–211	8	2.34	0.6103	0.5771447	(TCT)5

The sweetpotato accessions from Peru are genetically highly similar to accessions from Ecuador and clustered together along with the tetraploid accessions from Colombia (tetraploids) and germplasm from Venezuela. In addition, the accessions from Peru are in general clustered by ecological origin. Accessions from the coastal areas of Peru grouped together and the accessions from the highland forest clustered with material derived from Colombia with a similar ecology. Many of the Peruvian accessions could not be clearly differentiated from one another with these 20 SSR markers. These accessions have previously been noted as being highly similar (morphologically) suggesting the occurrence of genetic redundancy within the germplasm collection. Material from Africa, which has been thought of as a secondary center of diversity, was closely related to accessions from the Caribbean, Brazil, Argentina, and Paraguay. This suggests that sweetpotato may have moved from the eastern region of South America and the Caribbean to Africa. Reports have suggested this introduction was facilitated by the slave traders, although the dates of such transfers are unknown (Low et al., 2009).

The SSR data was loaded into R and analyzed with the polysat package (Clark and Jasieniuk, 2011) to calculate genetic diversity using allele frequencies. Heterozygosity was calculated by geographical region ranging from 0.78 to 0.8 with a mean of 0.792. The lowest level of heterozygosity was observed in the advanced breeding lines at 0.753 and the highest heterozygosity found in the landraces at 0.81. This difference could partially be due to the sample size bias since the landraces (4,255) were a much larger grouping than advanced breeding lines (236). However, typically landraces are more variable genetically than breeding lines. The Fst was also calculated by geographic origin and biological status. The Fst ranged from 0.000006 to 0.00223 among the different types of biological materials in this data set suggesting that interbreeding is occurring freely. The largest difference occurring among landraces and breeding lines. The geographical origin Fst ranged between 0.0009 and 0.017. The largest Fst values occurred between Peru or Ecuador compared with material from Africa and Asia and Oceania.

Since a high degree of similarity was observed among some of the accessions (Supplementary Figure 2), the data set was pruned to remove potential duplicates. All accessions that had ≥95% genetic similarity were removed from the data set and a new phylogeny containing 3,075 accessions was constructed (Supplementary Figure 3, Supplementary Table 1). Clustering patterns remained similar even when 48.5% of the accessions were removed from the data set. Removing highly similar accessions demonstrated that accessions are again generally grouped by country of origin. The germplasm from the Caribbean, Brazil, Argentina, Paraguay (eastern South America) was closely related and was similar genetically to material from Africa as observed in the phylogeny of all the germplasm (Figure 2 and Supplementary Figure 3).

**Figure 3:**
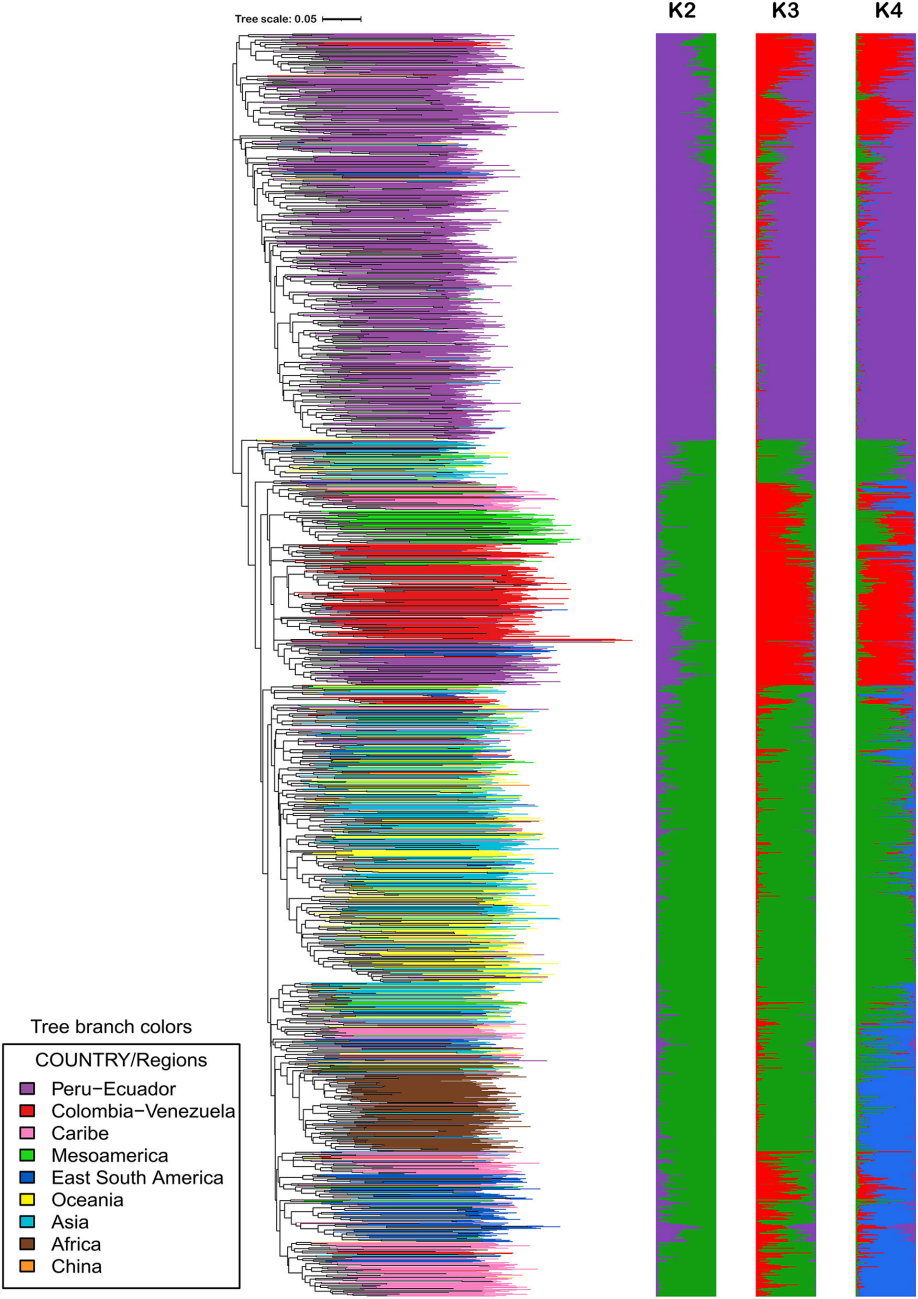
Analysis of population structure and phylogenetic relationships of 2,109 landraces in the germplasm collection at CIP. K = 2 to K = 4 is displayed here. The phylogeny was created from calculating a pairwise genetic similarity distance matrix and subsequently employing neighbor-joining to construct the intraspecific relationships. iTOL was employed to color code branches by country of origin or donations.

Since landraces typically tend to be highly variable compared to improved material and breeding lines and because the landraces represent the largest portion of accessions (71% or 4,255 accessions) in the germplasm collection at CIP, further analysis of only the landraces was carried out. The landraces were obtained from various geographic origins over many years. Accessions of landraces that were highly similar (95% or higher similarity) were once again removed from the data set. After the removal of genetically similar accessions, a total of 2,109 (49% of the total landraces) remained and these were subjected to phylogenetic and population structure analysis (Figure 3). The clustering patterns were similar to what was observed in all 5,979 accessions. Eliminating genetic redundancy demonstrated more prevalence of phylogeography within this collection.

The STRUCTURE results are presented in Figure 3. Support was obtained for both K = 2 and K = 4 depending on the method used to evaluate the number of populations (Evanno method or STRUCTURE statistical output) and/or the replicated STRUCTURE run chosen (Supplementary Figure 4). The authors of STRUCTURE state that while it may not be possible to know the true value of K, one should try to pick the smallest value that captures the major structure of the data (Pritchard et al., 2000). Wadl et al. (2018) evaluated part of the USDA sweetpotato germplasm collection of 417 accessions and reported peaks occurring at K = 2 and K = 4, similar to the results reported in Supplementary Figure 4. Although support was found for multiple K values, the authors chose K = 4 for the USDA germplasm collection because it represented the gene pools and historical movement of germplasm (Wadl et al., 2018).

In the CIP landrace collection, K = 2 groups the 2109 accessions from all regions (Africa, Asia, Caribbean, South America) into one population apart from accessions from Peru and Ecuador which are grouped into the second population. At this (2) K value, accessions from Mesoamerica and Peru/Ecuador separated supporting the hypothesis that there are a Northern and Southern gene pool (Roullier et al., 2011). At K = 4, the population is separated into accessions from: 1) Colombia and Venezuela, 2) Peru and Ecuador, 3) Asia, Oceania, Mesoamerica, and 4) Africa, the Caribbean and Eastern South America. Simulation studies have noted that uneven sampling among subpopulations lead to downward biased estimates of K by merging small subpopulations and extensive subpopulations were split despite their belongingness to a population (Puechmaille, 2016). Therefore, the data set was pruned to 1550 accessions in order to have more even sampling distributions among subpopulations (data not shown). This data reduction produced strong support for K = 4. A population number of K = 4 agrees with the number of major clades produced in the phylogeny and it represents the gene pools of sweetpotato.

Overall, the level of admixture among individuals in the CIP sweetpotato collection was lower than that observed in the CIP potato collection (Ellis et al., 2018). For example, the germplasm from Africa which includes a lot of breeding lines which tended to have low levels of genetic variation and also had low levels of admixture. The sweetpotato material from Peru had low gene flow suggesting it has been genetically isolated over time. Wadl et al. (2018) also reported lower levels of admixture among accessions from Central and South America but observed higher admixture among other accessions within the USDA genebanks - notably material from North America. Their data also supported the occurrence of a Northern and Southern gene pool as reported by Roullier et al., 2011. Somewhat higher levels of gene flow were observed in accessions from other geographic origins such as material from Oceania and Asia.

### Genetic Identity of the CIP Germplasm Collection

Genetic contamination or mixing of accessions can occur in any genebanks or similar program that routinely handles or manipulates large numbers of individually maintained samples. This is especially true when phenotypic variation is subtle (Girma et al., 2012; Bergelson et al., 2016). It is estimated that up to one-third of cell lines used in medical research may be contaminated or misidentified (Hughes et al., 2007). Although estimates of error in plant genebanks are not thought to be so high, SNP genotyping revealed approximately 5% error in a collection of *Arabidopsis* accessions (Anastasio et al., 2011). In another instances, an evaluation of the yam (*Dioscorea*) collection at the International Institute of Tropical Agriculture (IITA) revealed that 20.6% of 3,156 accessions were not true-to-type (nTTT) (Girma et al., 2012). Although errors have occasionally been reported or alluded to, the materials in few plant collections have been systematically assessed for true to type (TTT) due to resource availability and/or reputational stakes. Internal reports at CIP (unpublished) suggested that some of the clonal germplasm may have gotten mixed or mislabeled in the 30–40 years of maintenance and regeneration (Perazzo et al., 1999-2000). A total of 250 *in vitro* accessions of potato from the CIP collection was previously evaluated with a 12K SNP array that included an original mother plant and its *in vitro* counterpart in side by side comparisons. In that study, a 4.4% error level was detected (Ellis et al., 2018). The CIP sweetpotato collection however has not been systematically assessed, partially or on an entire collection wide basis. Complete removal of errors in any experiment or when handling large numbers of accessions day to day is unrealistic. If the average error rate in the mixing or mislabeling of accessions in a collection was only 1% per year, then as high as 30% of the total accessions could be incorrect after 30 years, assuming only one annual propagation cycle for *in vitro* plantlets.

**Figure 4:**
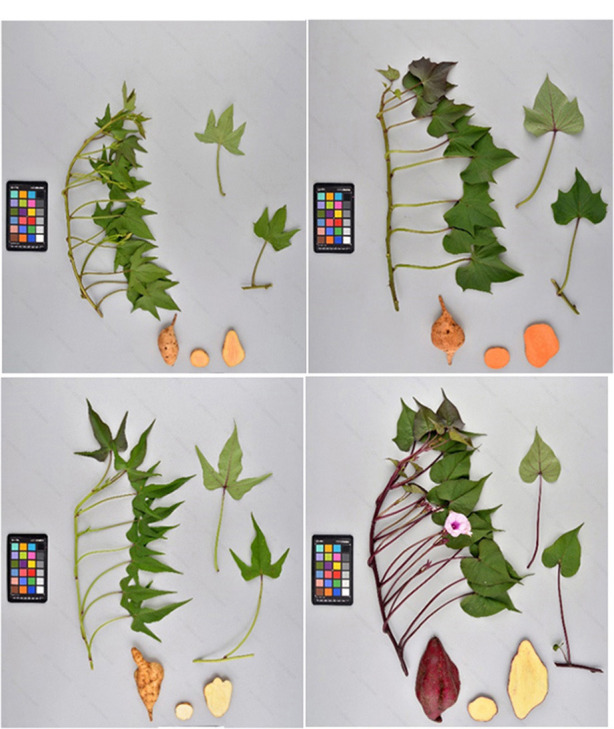
Examples of *in vitro* and mother plants with different phenotypes and genotypes of the same accession. Storage roots and leaves from greenhouse mother plants and *in vitro* derived plants from two accessions not true to type (nTTT) as observed by different phenotypes indicating a genetic mixture and storage roots. Upper left: CIP 420081 original source plant. Upper right: CIP 420081 *in vitro* derived plant. Lower left: CIP 420101 original source plant. Lower right: CIP 420101 *in vitro*-derived plant.

**Figure 5:**
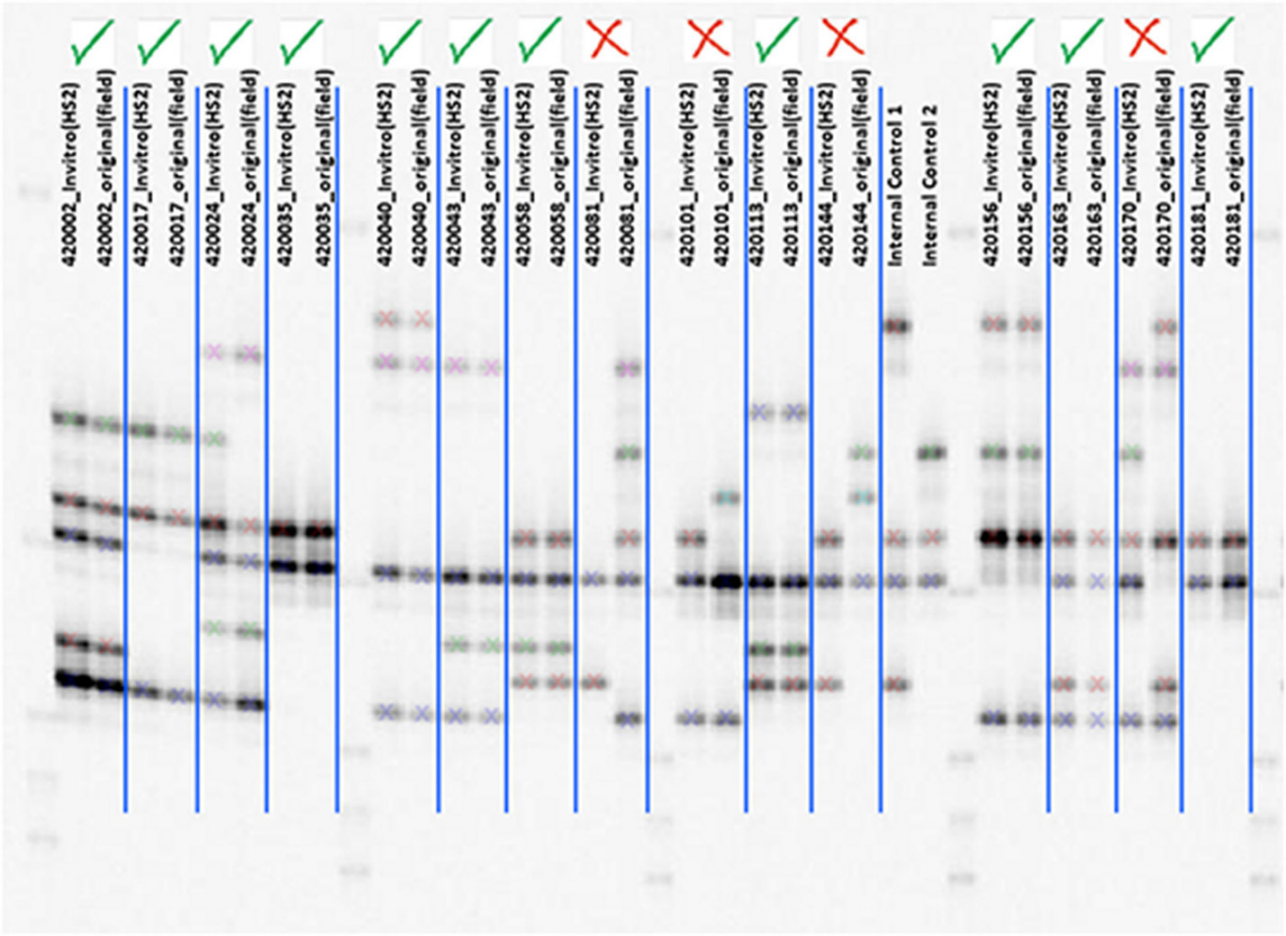
Checking clonal identity using marker IBE2 shows allele patterns of *in vitro* and mother plants with a green check indicating a match and red X indicating a mismatch.

Of the 5,979 accessions in the CIP collection, 2,711 (45.3%) currently have both an original mother plants maintained in the greenhouse, and an *in vitro* counterpart, both of which have been undergoing continual periodic propagation for several decades. These matched pairs are useful in efforts to detect errors in accession identification. In this study, SSR markers were used to genotype both the *in vitro* and mother plants of individual accessions (Figure 4). The resultant fingerprints from dual sources of the same accession provided a basis for detecting or confirming misidentifications (Figure 5). The SSR data revealed that in 533 accessions (19.6%) the allelic patterns obtained from the mother plant and the *in vitro* counterpart of the same accession did not match. In comparison, 80.4% did match and had identical fingerprint patterns. Extensive morphological characterization data, based on 30 standard genebanks descriptors in the field, was also carried out to confirm and validate which sample (mother plant or *in vitro* sample planted side by side in the field) matched the historical passport data and was the TTT individual (Figure 4). For example, the mother plant and *in vitro* sample of CIP 420101 had strikingly different root flesh colors and leaf morphologies. The TTT sample of accession 420101 (based on available passport data) had hastate leaves, deep leaf lobes, and dark yellow skin color matching the mother plant. In contrast, the *in vitro* sample had cordate leaves, slight leaf lobes, and dark red skin color and thus was nTTT. These two samples also did not cluster close together in the phylogeny further confirming they were not a match. The mother plants and *in vitro* sample of CIP accession 420081 also did not match based either on morphological data or SSR allelic banding patterns. In this accession (CIP 420081), the paired samples had different morphological features observed in the leaf veins, storage root skin color, and slight differences in leaf lobes (Figure 4). In both cases, original passport data support the mother plant as being the correct clones and the *in vitro* sample being incorrect. Thus, both accessions were reintroduced into *in vitro* and the collection using the mother plant.

Replacements of incorrectly identified clones to the *in vitro* collections is time-consuming and costly compared to replacing misidentified clones in field collections. In the latter case, this is readily accomplished by acclimating an *in vitro* plantlet, transferring it to the soil, and then hardening it off. Replacing a misidentified *in vitro* culture is more complicated in that it requires the re-introduction of the accession to aseptic conditions and pathogen elimination to confirm a disease-free status. This process can take 2 to 4 years at CIP. During the re-introduction process, the material cannot be shared externally without certifications that the material is free of known pathogens. If the clone should test negative for pathogens, the process is somewhat shorter, but this occurs infrequently. Longer periods may be necessary due to existing backlogs of material that also require pathogen elimination and introduction to *in vitro* maintenance. Oftentimes, a bottleneck in this process is the absence of adequate greenhouse space for the biological indexing and the facilities and resources required for thermotherapy and meristem isolation. Putatively virus-free material is subjected to the second round of virus testing and indexing to ensure the thermotherapy or meristem isolation process was effective. This adds to the total time required to produce virus-free plantlets.

Many (80.4%) of the paired (mother plant and *in vitro* counterpart) samples from the same accessions had similar allelic profiles along with morphological similarity. CIP 420017 and CIP 420156 (data not shown) were true to type (TTT) having matching root and leaf morphologies along with the other descriptors data which matched the original passport data. These accessions also had to match allelic profiles based on the 20 SSR markers. Simultaneous maintenance of paired samples enabled the corrections of these misidentifications. Although the pairs were not available for every accession, 45% of the collection was comprehensively evaluated for genetic identity, and accessions were corrected as errors were detected based on the genotyping and phenotyping data. Genotyping and phenotyping were also performed for accessions without paired samples, although errors in these accessions were more difficult to discern from historical data in the absence of complete phenotyping data. Interestingly, although the examples in Figure 4 show errors in the *in vitro* sample, errors were detected both within *in vitro* and the mother plant or greenhouse collections (All data shown previously on evaluating the entire germplasm collection utilized the sample determined to be true to type sample for each accession prior to the analysis).

### Reconciling Accession Identities Between Germplasm Collections

A subset of 189 accessions from the USDA sweetpotato collection (https://npgsweb.arsgrin.gov/gringlobal/search, Supplementary Table 2) were evaluated with the same 20 SSR markers used to examine the CIP germplasm (Tables 1, 2). These included 91 accessions (48%) that were known to have been transferred or donated from one collection to the other (USDA to CIP, or vice versa) between 1988 and 1990 and thus were chosen to evaluate the maintenance of genetic integrity over time between separate locations. The number of alleles per marker in the accessions from the USDA collection ranged from 3 to 20 with an average number of 11.2 per locus. Two major clades were observed in the phylogenies of the USDA material (Supplementary Figure 5). The branch lengths between these clades were short suggesting low divergence among these groupings or the presence of some hybrids. Unlike the CIP accessions, fewer of the accessions from the USDA collection were distinguishable from one another using the 20 SSR markers. This is likely due to the much smaller sample size when compared with the CIP collection and the fact that the USDA collection is known to contain many heirloom varieties with similar pedigrees.

**Figure 6:**
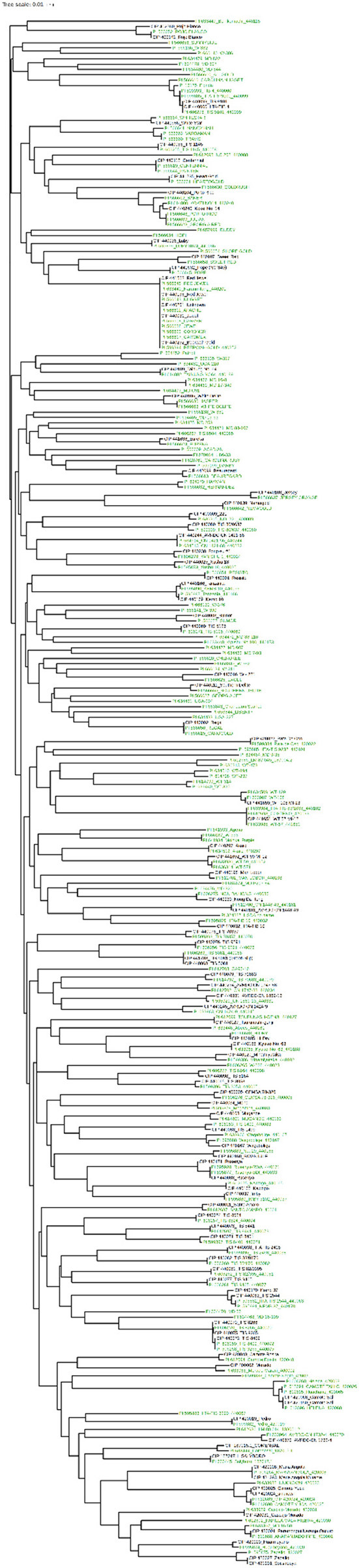
Comparison of the 100 most similar CIP accessions (black) to the 189 USDA (green) accessions. The phylogeny was created from calculating a pairwise genetic similarity distance matrix and subsequently employing neighbor-joining to construct the intraspecific relationships. iTOL was employed to color code branches.

Apparent redundancies were, however, identified within this group of materials. For example, the accessions PI 566618 (cv. Carver), PI 566648 (cv. Red Jewel), PI 566611 (cv. Apache), PI 566643 (cv. Nugget), PI 599394 (cv. Beerwah Gold), and PI 566617 (cv. Caromex) formed a single cluster. Several of these accessions have cv. Jewel in their pedigree or were a selection from cv. Jewel. This is reflected in their clustering. Other putative duplicates only occurred in pairs and at times the accessions in these pairs had similar names such as PI 634466 MD 16-8 and PI 634469 MD 17-340 or PI 634514 CN 1421-56 and PI 634513 CN 1421-68. These are breeding lines and likely selections from the same families within breeding programs. Hence, they are genetically highly similar. The 189 USDA accessions were also analyzed by comparing them to the entire CIP collection by producing a phylogeny from the marker data (data not shown) to reconcile the identification of the accessions shared between these collections and to verify the identity of exchanged materials (unpublished data). A phylogeny was constructed that included 100 of the CIP accessions most closely related to the 189 accessions from the USDA collection (Figure 6, Supplementary Table 2). Most of the common accessions genotypically matched their counterpart. However, some accessions (16/91 or 17.5%) did not match as expected, although they had been previously passed directly from one genebanks to another. In such instances, it proved difficult to determine which identification was correct, and errors were identified in both collections. A portion of the remaining USDA accessions (33) had names and passport data similar to accessions in the CIP collection, but had been acquired independently (not passed between USDA or CIP genebanks). Due to inadequate documentation, it was not always possible to determine if similarly named clones were supposed to be the same. However, the marker data revealed that 20 of the 33 accessions were genetically similar while the remaining 13 accessions had the same accession names, but different genotypes. This suggested that these accessions either were incorrectly documented or mis-identified when acquired, became mixed over time, or that two distinct varieties were given the same name independently, which frequently occurs. The 65 remaining USDA accessions (34.3%) were unique and did not cluster closely with any of the CIP accessions. These 65 accessions represent unique material and, as such, are likely future additions to the CIP sweetpotato collections. These results, overall, serve to emphasize the importance of timely and thorough documentation of newly acquired germplasm as a hedge against potential future problems with accession identification and indicate that genotyping at the time of introduction can play an important role in the long-term maintenance of plant materials.

## Concluding Remarks

The entire *in trust* germplasm collection of cultivated sweetpotato, containing 5,979 accessions, was characterized using 20 SSR markers. Phylogenetic relationships were calculated revealing intraspecific relationships on a collection-wide basis and identified likely genetic redundancies within the germplasm collection, especially among the accessions from Peru. Phylogeographic signals were observed for the majority of accessions, which were mainly grouped by country of origin. Population structure analysis revealed some admixtures in the collection, but many accessions were homogenous and did not show any introgressions from the other populations. The structure data indicated the presence of few hybrids.

Identity errors were detected in the collection by evaluating *in vitro* and mother plants, side by side. Identifying and sorting out errors can be challenging, costly, and time-consuming. Yet, identifying these errors is necessary to ensure that users obtain the clone they need, with the required traits, based on previously collected data and to reduce long-term costs associated with maintenance of the collection. In general, users expect the material they receive from a genebanks to match phenotypic data either previously published or stored in the genebanks database. Reconciling accession identifications in sweetpotato germplasm collections *via* genotyping is useful and a necessary approach to reveal genetic gaps, identification errors, and duplications. This rationalization can also serve as a safety backup if duplicates are discovered or if materials are lost. As far as the authors are aware, this is the first report on genotyping and confirming the genetic identity of an entire genebanks collection in sweetpotato and the first to attempt to reconcile accession identifications across collections.

## Data Availability Statement

The SSR data from this work are included in Supplementary Table 3; further inquiries can be directed to the corresponding author.

## Author Contributions

NA and DE designed and directed the study, managed the data analysis, and drafted the manuscript. RR and RA performed DNA extractions, PCR reactions, LiCor gel separations, assisted with marker selection\screening, and edited the manuscript. RR and GR assisted with data analysis, preparation of figures and tables, and edited the manuscript. NM supervised and provided guidance on data analysis and edited the manuscript. AP provided *in vitro* material for the study, coordinated sample handling and tracking, and edited the manuscript. RJ provided DNA of USDA material and assisted with drafting and editing of the manuscript. All authors contributed to the article and approved the submitted version.

## Conflict of Interest

The authors declare that the research was conducted in the absence of any commercial or financial relationships that could be construed as a potential conflict of interest.

## Publisher's Note

All claims expressed in this article are solely those of the authors and do not necessarily represent those of their affiliated organizations, or those of the publisher, the editors and the reviewers. Any product that may be evaluated in this article, or claim that may be made by its manufacturer, is not guaranteed or endorsed by the publisher.

## Supplementary Material

Supplementary Figure 1 Principal Component Analysis (PCoA) of entire sweetpotato collections (5,979 accessions). Country of origin or donation country color code: Peru = purple. Red = Venezuela and Colombia. Green = Mexico and Central America. Pink = Caribbean. Africa = brown. USA = black. Blue = Argentina, Paraguay, and Brazil. Turquoise = Asia. Yellow = Oceania.

Supplementary Figure 2 A plot of pairwise genetic distances of all 5,979 accessions showing a high number of genetic duplicates in the collection. The Y-axis contains the calculated genetic distance between individuals with many pairwise combinations having 0 genetic distance.

Supplementary Figure 3 Neighbor-joining phylogeny of accessions with ≤ 95% similarity effectively removing potential duplicates or highly related samples. A total of 3,075 accessions were used to construct the dendrogram. The iTOL was employed to color code branches by country of origin or donation. Accessions from Peru are colored in purple, red are accessions from Venezuela and Colombia, Mexico, and Central America are colored green, Caribbean are colored in pink, Africa is colored brown, USA is black, blue is Argentina, Paraguay, and Brazil, turquoise is Asia, and yellow is Oceania.

Supplementary Figure 4 Delta K values for different numbers of populations in a STRUCTURE analysis.

Supplementary Figure 5 Phylogeny of 189 USDA sweetpotato accessions. The phylogeny was created from calculating a pairwise genetic similarity distance matrix and subsequently employing neighbor joining to construct the intraspecific relationships.
