# Supplementary figures and images for: Genetic Identity, Diversity, and Population Structure of CIP's Sweetpotato (I. batatas) Germplasm Collection
Source: Front Plant Sci. 2021 Oct 28;12:660012. doi: 10.3389/fpls.2021.660012 (PMC8589021; doi:10.3389/fpls.2021.660012)

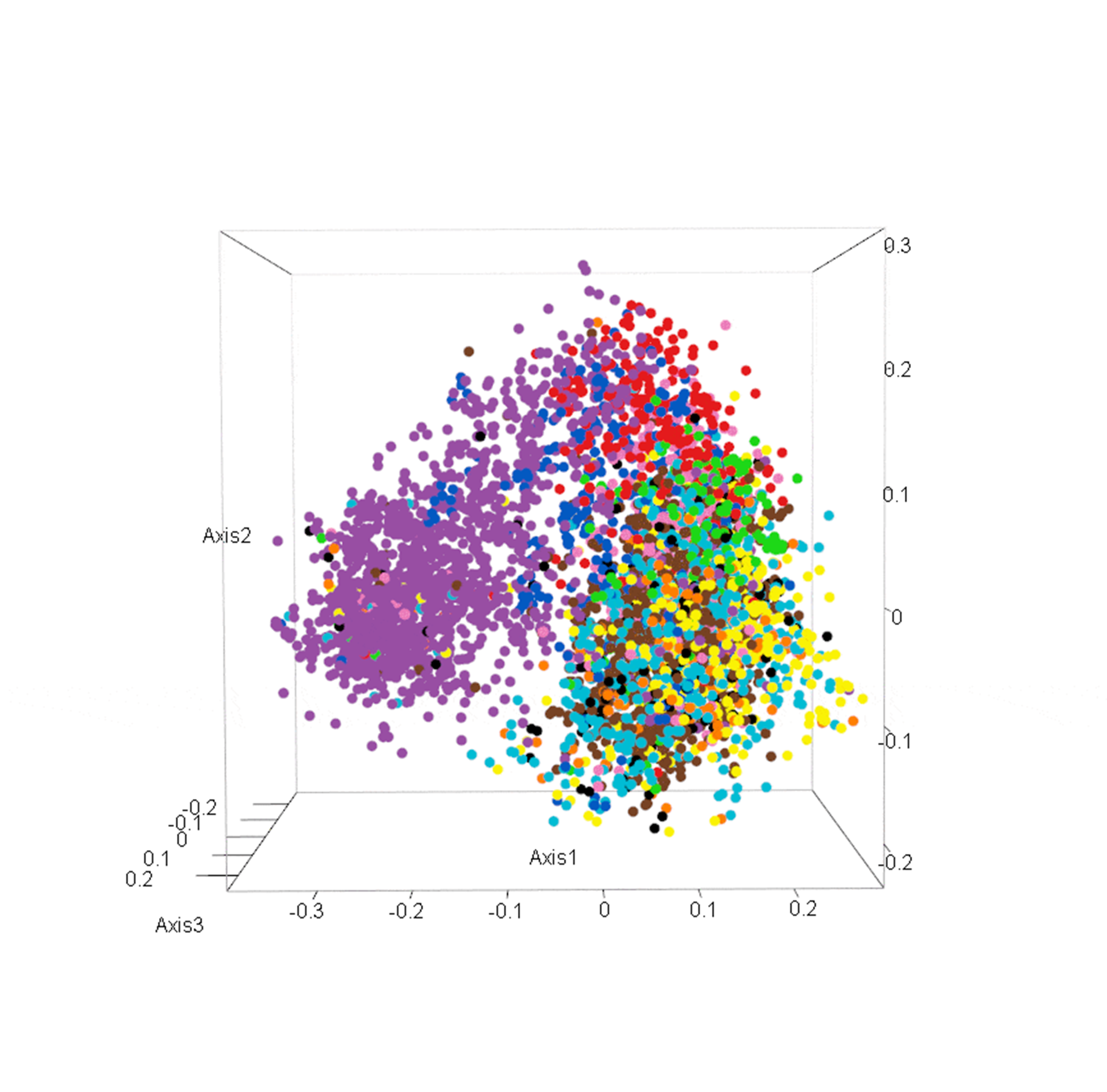

Supplement: Supplementary Figure 1 — Principal Component Analysis (PCoA) of entire sweetpotato collections (5,979 accessions). Country of origin or donation country color code: Peru = purple. Red = Venezuela and Colombia. Green = Mexico and Central America. Pink = Caribbean. Africa = brown. USA = black. Blue = Argentina, Paraguay, and Brazil. Turquoise = Asia. Yellow = Oceania. [file Image_1.TIF]

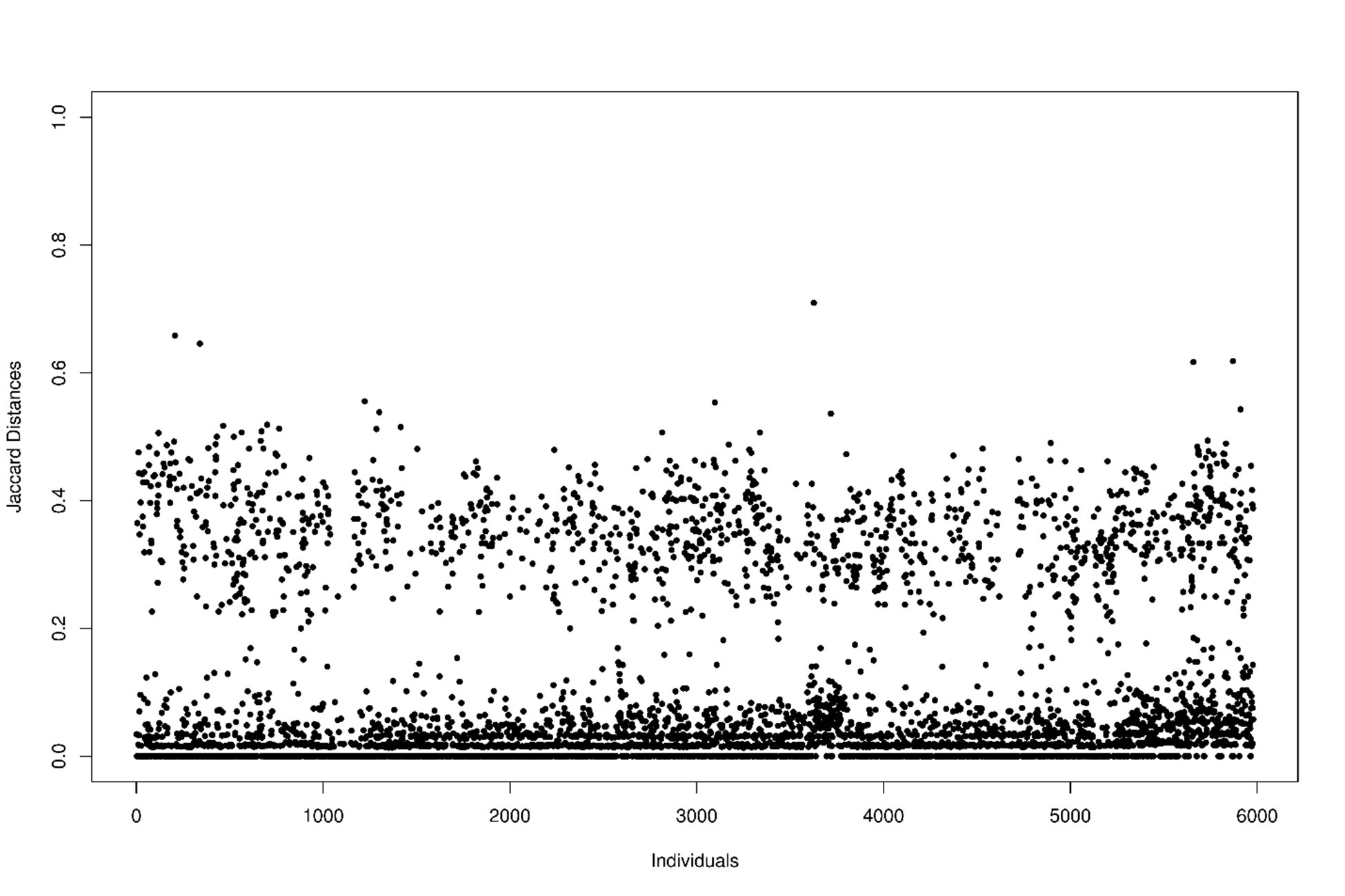

Supplement: Supplementary Figure 2 — A plot of pairwise genetic distances of all 5,979 accessions showing a high number of genetic duplicates in the collection. The Y-axis contains the calculated genetic distance between individuals with many pairwise combinations having 0 genetic distance. [file Image_2.TIF]

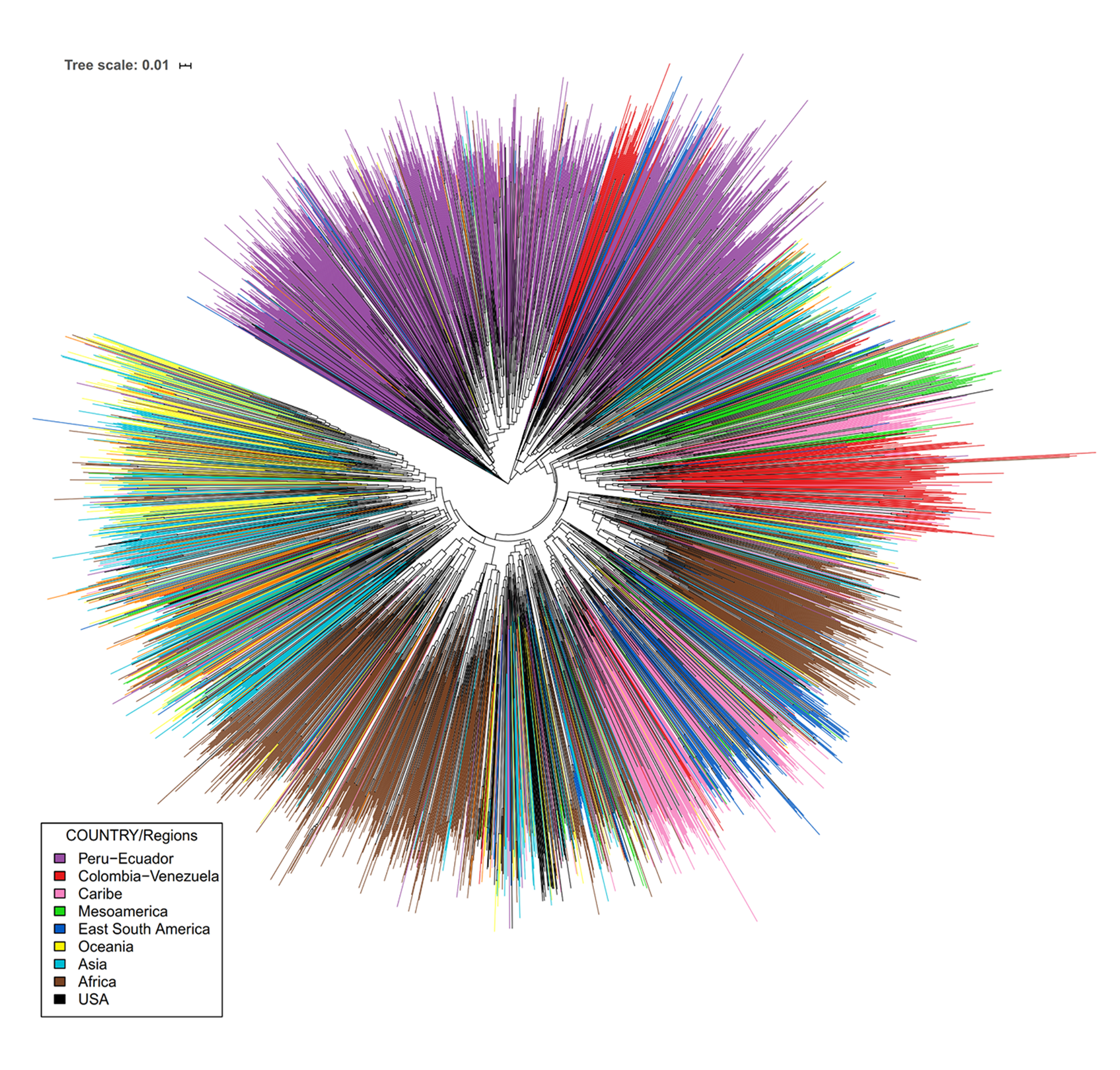

Supplement: Supplementary Figure 3 — Neighbor-joining phylogeny of accessions with ≤ 95% similarity effectively removing potential duplicates or highly related samples. A total of 3,075 accessions were used to construct the dendrogram. The iTOL was employed to color code branches by country of origin or donation. Accessions from Peru are colored in purple, red are accessions from Venezuela and Colombia, Mexico, and Central America are colored green, Caribbean are colored in pink, Africa is colored brown, USA is black, blue is Argentina, Paraguay, and Brazil, turquoise is Asia, and yellow is Oceania. [file Image_3.TIF]

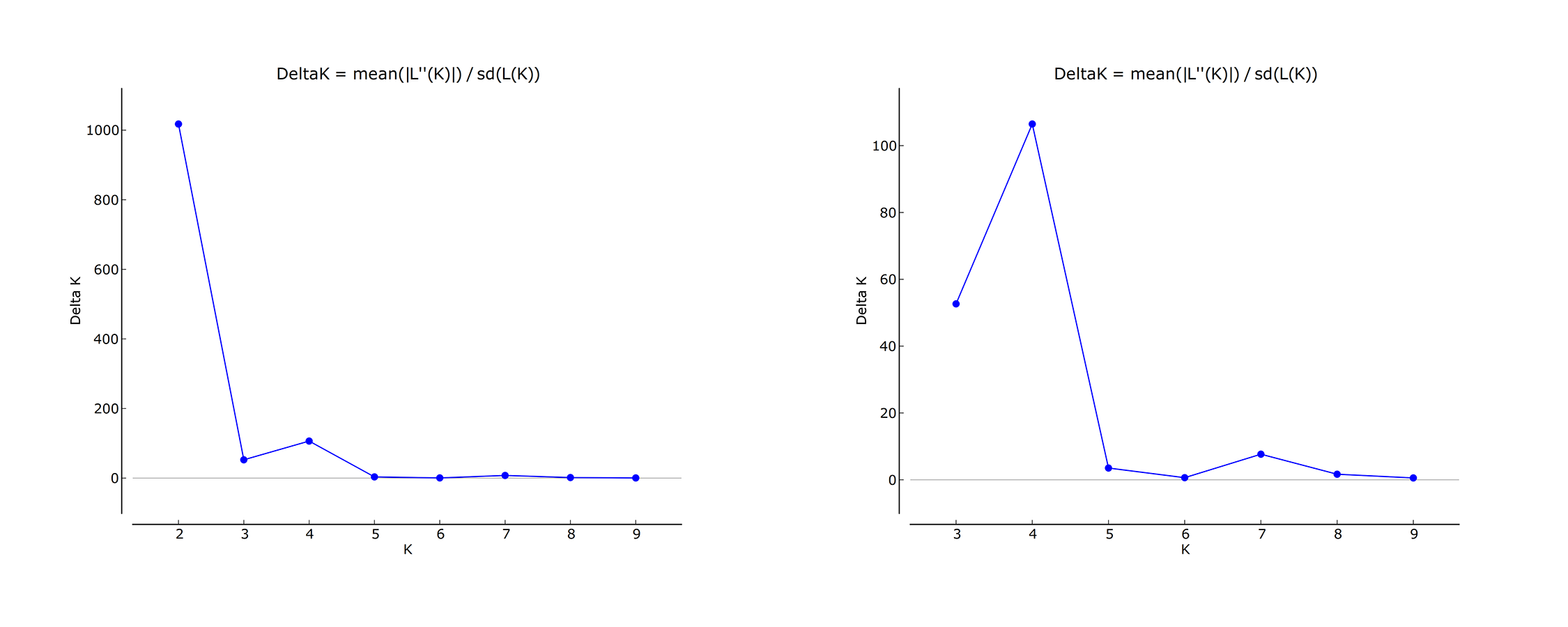

Supplement: Supplementary Figure 4 — Delta K values for different numbers of populations in a STRUCTURE analysis. [file Image_4.TIF]

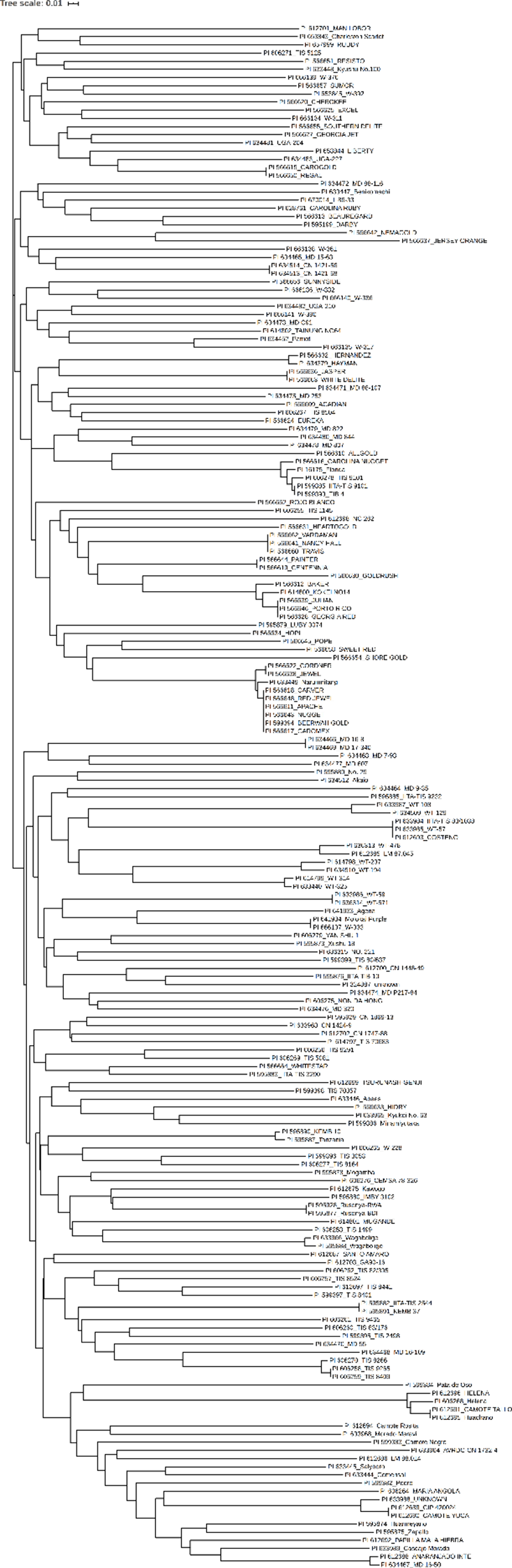

Supplement: Supplementary Figure 5 — Phylogeny of 189 USDA sweetpotato accessions. The phylogeny was created from calculating a pairwise genetic similarity distance matrix and subsequently employing neighbor joining to construct the intraspecific relationships. [file Image_5.TIF]
